# Severe headache as a key symptom in bilateral idiopathic adrenal hyperplasia-induced primary hyperaldosteronism: A case report: Retraction

**DOI:** 10.1097/MD.0000000000044339

**Published:** 2025-08-22

**Authors:** Ali Safaa Abduljabbar, Hashim Talib Hashim, Ahmed Dheyaa Al-Obaidi, Ashraf Fhed Mohammed Basalilah, Israa Al-gburi, Zaid Saad Abed Madhi

The article “Severe headache as a key symptom in bilateral idiopathic adrenal hyperplasia-induced primary hyperaldosteronism: A case report”^1^, which appears in Volume 104, Issue 16 of *Medicine*, is being retracted. Information brought to our attention during a recent investigation casts doubt on both the authorship and credibility of the presented case, and the Publisher has lost faith in the integrity of the content.
